# Voluntary wheel exercise reduces pain behavior by modulating neuroinflammation in neuropathic pain

**DOI:** 10.3389/fnmol.2026.1832751

**Published:** 2026-06-12

**Authors:** Yuqi Zhu, Wenyang Xu, Jinhui Zhang

**Affiliations:** 1 School of Economics and Management, Shanghai University of Sport, Shanghai, China; 2 School of Exercise and Health, Shanghai University of Sport, Shanghai, China; 3 Department of Physical Education, Xidian University, Xi'an, China

**Keywords:** analgesic effect, analgesic mechanism, neuroinflammation, neuropathic pain, voluntary wheel running

## Abstract

Neuropathic pain (NP) is a common condition caused by injury or disease of the somatosensory nervous system. It is associated with excessive inflammation of the central and peripheral nervous system. The treatment of NP is extremely challenging. In recent years, exercise therapy has received extensive research attention. Voluntary wheel running (VWR) is a simple aerobic exercise commonly used in animal experiments. VWR-induced analgesia seems to be associated with re-establishment of the neuroinflammatory system. This paper discusses the effects of VWR on the inflammatory response of the nervous system, including the brain, spinal cord, dorsal root ganglia (DRG), and peripheral nerves involved in NP regulation. VWR works by decreasing pro-inflammatory cytokines in the brain, spinal cord, DRG, and peripheral nerves, as well as by increasing anti-inflammatory cytokines to show anti-inflammatory effects. VWR also decreases the expression of glial cells in the brain, spinal cord, and DRG and modulates neuroinflammation. This study reviews the mechanisms by which VWR modulates neuroinflammation in NP to reduce pain behaviors and improve our understanding of the VWR role in NP treatment.

## 1 Introduction

Neuropathic pain (NP) is caused by injury or disease of the somatosensory nervous system, and its core symptoms usually include spontaneous pain, hyperalgesia, allodynia, and paresthesia (Inoue and Tsuda, 2018). According to epidemiological surveys, the incidence of NP in the general population is approximately 6.9–10% (Van Hecke et al., 2014). NP is difficult to treat because it differs from nociceptive pain and often requires multimodal treatment (Finnerup et al., 2015; He et al., 2024). Non-pharmacological interventions, including exercise therapy, have therefore become an important direction in pain research (Leitzelar and Koltyn, 2021; He et al., 2024; Zheng et al., 2024). Neuroinflammation refers to inflammatory responses in the peripheral nervous system (PNS) and central nervous system (CNS) (Ji et al., 2014; Loureiro et al., 2025). It is characterized by glial activation and the production of inflammatory mediators that can amplify nociceptive signaling (Julius and Basbaum, 2001; Loureiro et al., 2025). The state of NP is closely associated with inflammatory responses that promote the development and persistence of pain (Ellis and Bennett, 2013). Clinical and animal studies indicate that exercise can modulate neuroinflammation and reduce NP-related behavior (Sheahan et al., 2015). Voluntary wheel running (VWR) is a simple aerobic exercise model in which mice run spontaneously on a wheel placed in the home cage. Unlike forced exercise models that may use aversive stimuli, VWR allows voluntary locomotion under relatively low-stress conditions (Goh and Ladiges, 2015; Manzanares et al., 2018). These characteristics make VWR suitable for long-term studies of exercise-induced analgesia in NP (Ye et al., 2018).

Previous studies have reported that VWR can regulate neuroinflammation and improve pain behavior in NP models (Benson et al., 2015; Grace et al., 2016; Sabharwal et al., 2016; Ye et al., 2018; Sánchez-Ventura et al., 2021). However, the mechanisms by which VWR alleviates NP have not been systematically summarized. This review aims to examine how VWR regulates neuroinflammation across the brain, spinal cord, dorsal root ganglia (DRG), and peripheral nerves, and to discuss potential upstream molecular pathways that may connect voluntary exercise with analgesic effects.

## 2 Effects of voluntary wheel running on neuropathic pain

Physical exercise can ameliorate chronic pain and NP in several preclinical and clinical scenarios (Dobson et al., 2014; Guo et al., 2019). A comprehensive review of the available literature indicates that VWR improves pain-related behavior and reduces pain sensitivity in animal models of NP. These benefits may involve multiple mechanisms, including attenuation of inflammatory responses, enhancement of neural plasticity, and reduction of anxiety-related behavior (Duman et al., 2008). Existing studies commonly use chronic constriction injury (CCI), partial sciatic nerve ligation (PSL), spinal cord injury (SCI), chemotherapy-induced peripheral neuropathy (CIPN), or drug-induced neuropathy models. Grace et al. and Green-Fulgham et al. used 6-week VWR preconditioning before CCI surgery in rats and found that mechanical hypersensitivity was alleviated after surgery (Grace et al., 2016; Green-Fulgham et al., 2022). Minami et al. used a PSL model and showed that active VWR induced exercise-related hypoalgesia in mice, possibly through reduced fear- and anxiety-related responses (Minami et al., 2023). Kami et al. also used the PSL model and found that VWR mice had longer withdrawal latency and earlier recovery toward preoperative levels than sedentary mice (Kami et al., 2020). Ye et al. established an antiretroviral therapy model by injecting 2,3-dideoxycytidine (ddC) into mice, followed by a 13-week VWR intervention; from the ninth week, VWR mice showed lower mechanical and thermal sensitivity than sedentary mice (Ye et al., 2018). Slivicki et al. used a paclitaxel-induced cancer pain model and found that VWR delayed the onset of abnormal pain and mitigated pain behavior when applied before, during, or after model establishment (Slivicki et al., 2019). Sánchez-Ventura et al. divided SCI mice into sedentary, enriched-environment, forced treadmill, and VWR groups. By the fourth week, the active groups had a lower proportion of mice with thermal hyperalgesia, and only the VWR group showed significantly improved Basso Mouse Scale (BMS) scores (Sánchez-Ventura et al., 2021).

One study examined VWR parameters in detail (Green-Fulgham et al., 2022). Running distance and running speed varied substantially among individual rats and between sexes, but these differences were not clearly correlated with sensory thresholds or hyperalgesia. In contrast, Kami et al. and Minami et al. reported a positive association between total running distance and withdrawal latency in VWR mice (Kami et al., 2020; Minami et al., 2023). The timing and duration of VWR also appear to influence outcomes. Preconditioning for less than 3 weeks may be insufficient to protect rats from NP, whereas the preventive benefits of VWR become evident between the third and sixth weeks (Green-Fulgham et al., 2022).

Forced exercise and voluntary exercise may involve distinct mechanisms, and voluntary exercise often shows stronger effects than forced exercise. This difference may be related to the stress induced by forced exercise, which can confound pain perception (Butler and Finn, 2009). Voluntary exercise also occurs during active waking phases and avoids sleep disruption caused by compulsory exercise regimens. In one SCI mouse study, the VWR group displayed greater activity than the forced treadmill group, including longer duration and greater distance covered (Sánchez-Ventura et al., 2021). Table 1 summarizes VWR interventions in animal models of NP.

**Table 1:** General characteristics of studies on voluntary wheel running for neuropathic pain.

Model	Sampling site	Study grouping	Duration of intervention	Pain indicators	Related cytokines/protein/ion	Main result	Reference
ART-induced mice (ddC- injection)	L3-L5 DRG	①Running/Saline (*n* = 18) ②No Running/ddC (*n* = 18) ③Running/ddC (*n* = 18)	13 weeks	Mechanical allodynia; Thermal nociception	TRPV1	Voluntary wheel running attenuated mechanical and thermal sensitivity after chronic ddC treatment	Ye et al. (2018)
PSL mice	brain sections	①Naive/Sedentary (*n* = 6) ②Naive/Runner (*n* = 6 ~ 8) ③Sham/Sedentary (*n* = 6) ④Sham/Runner (*n* = 6 ~ 8) ⑤PSL/Sedentary (*n* = 6 ~ 8) ⑥PSL/Runner (*n* = 6 ~ 10)	4 weeks	Heat hyperalgesia	latBA-Glu; medBA-Glu; CeA-GABA	The pain behaviors in NPP model mice were significantly improved by the VR protocol employed	Kami et al. (2020)
CCI rat	sciatic nerves; L4/5 DRG; L4/5 spinal cords	①Sham/ Sedentary ②Sham/ Run ③CCI/ Sedentary ④CCI/ Run	6 weeks	Mechanical allodynia	Nrf2	Voluntary running preconditioning attenuates subsequent neuropathic pain	Green-Fulgham et al. (2022)
CCI rat	L4-L5 dorsal quadrants and DRGs	①Sham/Locked wheel ②Sham/Running wheel ③CCI/Locked wheel ④CCI/Running wheel	6 weeks	Mechanical allodynia	IL-1β; GLT-1; NLRP3; NF-κB p65; BDNF; Iba1; CCL-2; ATF3; iNOS; Arg-1	Prior voluntary exercise persistently attenuated the severity of allodynia in a rat model of neuropathic pain	Grace et al. (2016)
CIPN mice	brain sections (hippocampus)	①Paclitaxel/ Exercise (*n* = 5/6) ②Paclitaxel/ Sedentary (*n* = 5/6) ③Vehicle/ Exercise (*n* = 5/6) ④Vehicle/ Sedentary (*n* = 5/6)	4 weeks	Mechanical allodynia; Cold stimulation	Ki67-; BrdU-	Voluntary running attenuates the development of paclitaxel-induced allodynia; prior running wheel activity partially delays the onset of paclitaxel-induced allodynia; running wheel activity partially alleviates already established paclitaxel-induced hypersensitivities	Slivicki et al. (2019)
PSL mice	brain sections	①Naive-Sedentary mice (*n* = 9) ②Sham-Sedentary mice (*n* = 9) ③PSL-Sedentary mice (*n* = 9) ④Naive-Runner mice (*n* = 9) ⑤Sham-Runner mice (*n* = 9) ⑥PSL-Runner mice (*n* = 10)	4 weeks	Heat hyperalgesia	FosB^+^; GABAergic interneurons	VR for 2 weeks before and after PSL surgery significantly improved pain behaviors on Post 14 days; more active VR can enhance the EIH effect	Minami et al. (2023)
SCI mice	T11 spinal cord; L4-5 spinal cords; brainstem	①Uninjured control (*n* = 5) ②Sedentary (*n* = 5) ③Enriched environment (*n* = 6) ④Forced treadmill running (*n* = 5) ⑤Voluntary wheel (*n* = 7)	4 weeks	Thermal algesimetry	KCC2	Voluntary wheel running was able to modulate both hyperreflexia and hyperalgesia.	Sánchez-Ventura et al. (2021)

VWR, voluntary wheel running; VR, voluntary running; NP/NPP, neuropathic pain; ART, antiretroviral therapy; ddC, dideoxycytidine; latBA-Glu, lateral basal amygdala-glutamate; medBA-Glu, medial basal amygdala-glutamate; CeA-GABA, central nuclei of amygdala-gamma-aminobutyric acid; PSL, partial sciatic nerve ligation; CCI, chronic constriction injury; BDNF, brain-derived neurotrophic factor; CIPN, chemotherapy-induced peripheral neuropathy; SCI, spinal cord injury.

## 3 Mechanisms of VWR for NP by modulating neuroinflammation

**Figure 1:**
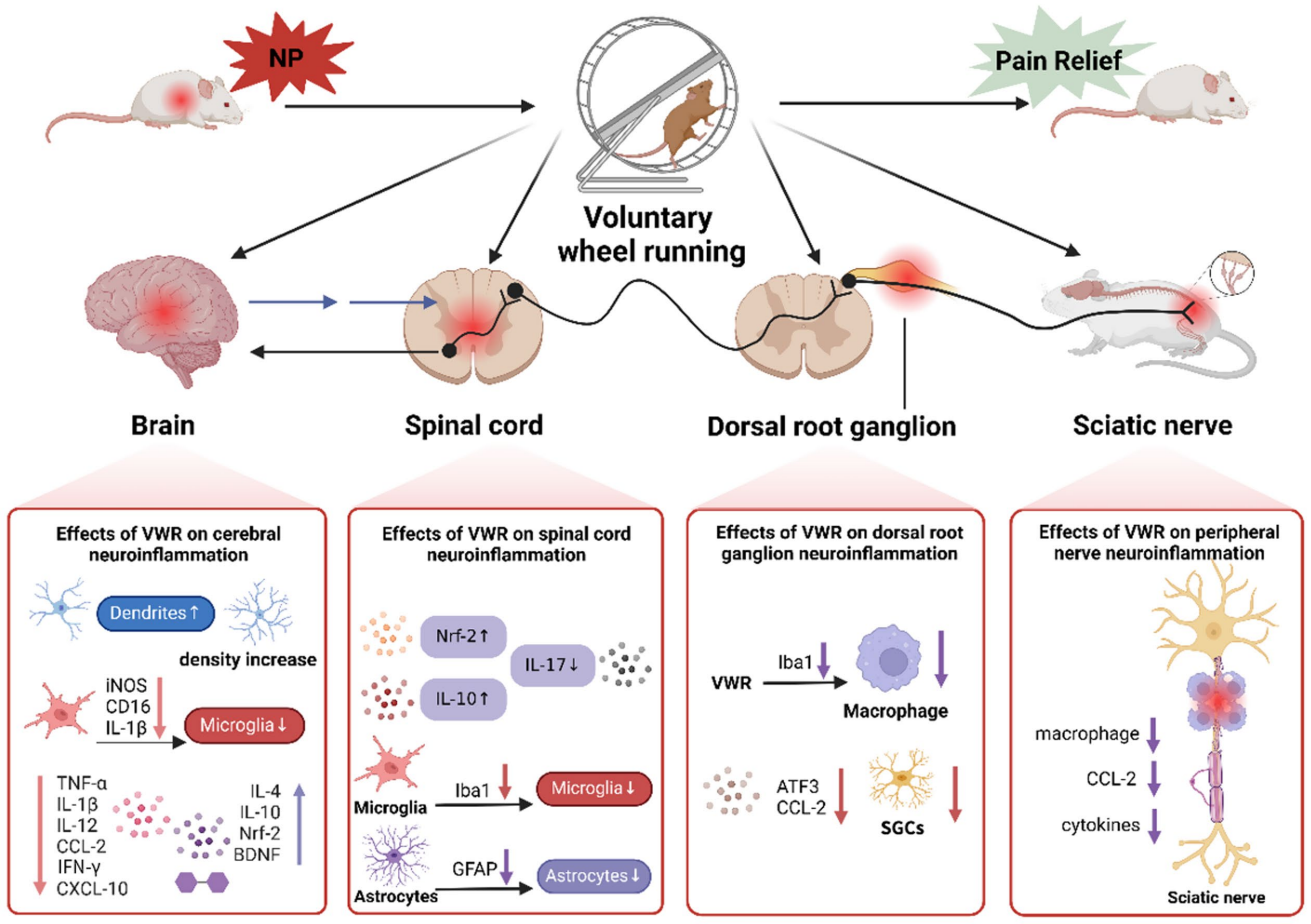
The mechanisms involved in the relief of neuropathic pain by voluntary wheel running. Created with BioRender.com. VWR, voluntary wheel running; IL-17, interleukin-17; IL-10, interleukin-10; IL-6, interleukin-6; IL-4, interleukin-4; IL-1β, interleukin-1β; BDNF, brain-derived neurotrophic factor; TNF-α, tumor necrosis factor-α; IFN-*γ*, interferon-γ; Nrf2, nuclear factor E2-related factor 2; ATF3, activating transcription factor 3; CCL-2, C-C motif chemokine ligand 2; GFAP, glial fibrillary acidic protein; SGCs, satellite glial cells; DRG, dorsal root ganglion; NP, neuropathic pain.

Peripheral nerve injury and disease often result in pain that persists beyond the resolution of the initial injury, suggesting an active disease-promoting process that contributes to NP (Sommer et al., 2018). Neuroinflammatory responses are triggered by peripheral inflammation and can lead to synaptic dysfunction, neuronal damage, and lesions involving the brain, spinal cord, DRG, and peripheral nerves (Kitazawa et al., 2005; Lyman et al., 2014; Bairamian et al., 2022). Neuroinflammation has multiple regulators, including macrophages, cytokines, and chemokines; in the spinal cord and brain, microglia and astrocytes are especially important (Sommer et al., 2018). Pro-inflammatory cytokines and chemokines increase after nerve injury and further stimulate inflammatory signaling. Tumor necrosis factor-*α* (TNF-α) is a typical inflammatory mediator, and cerebral ischemic injury can trigger apoptotic signaling through TNF-α receptor 1 (TNFR1) (Watters and O’Connor, 2011). Anti-inflammatory cytokines such as interleukin (IL)-10 and IL-4 can limit tissue injury and restore immune balance (Clark et al., 2013). Thus, inflammation and NP interact bidirectionally through cytokine-driven regulation of neuronal excitability and pain-signal transduction. VWR increases anti-inflammatory cytokines and decreases pro-inflammatory cytokines and glial activation (Grace et al., 2016; Feldman-Goriachnik et al., 2022), suggesting that VWR may alleviate NP by coordinating inflammatory and neuroplastic changes across multiple anatomical levels (Figure 1).

### 3.1 Effects of VWR on cerebral neuroinflammation

Neuroinflammation may lead to cortical and hippocampal dysfunction, resulting in NP (Spielman et al., 2017; Reguilón et al., 2020). VWR can reduce the accumulation of neuroinflammatory factors in the cortex and hippocampus (Hu et al., 2025). Several studies indicate that VWR regulates inflammatory-factor expression and prevents brain neuroinflammation induced by complex stressors or injuries (Benson et al., 2015; Reguilón et al., 2020). VWR can also affect hypothalamic neuroinflammation through changes in pro-IL-18, IL-1β, and IL-18 expression (Grabowski et al., 2025). After 7 weeks of VWR in C57BL/6 mice, anti-inflammatory cytokines IL-4 and IL-10 increased in the frontal cortex, hippocampus, and cerebellum (Spielman et al., 2017). IL-10 plays an important role in NP improvement by reducing inflammatory mediators such as TNF-*α*, IL-1β, IL-6, IL-8, IL-12, and IL-23 (Kwilasz et al., 2015). In another experiment, voluntary wheels were placed in the cages of SNI rats within an enriched environment (Gong et al., 2018). After 45 days, pain behavior improved, TNF-α and IL-1β levels in the brain decreased, and IL-10 expression increased. However, this study cannot directly isolate the contribution of VWR from environmental enrichment, and further work is needed. Chemokines are also important neuroinflammatory mediators. Carlin et al. showed that VWR can inhibit overexpression of the pro-inflammatory chemokines CCL-2 and CXCL-10, thereby reducing brain neuroinflammation (Carlin et al., 2016). In a recent traumatic brain injury study, 7 days of VWR significantly alleviated brain inflammatory responses, with decreased levels of IL-12, interferon-*γ* (IFN-γ), and CCL-2 (Hu et al., 2023).

Glial activation is a major driver of brain neuroinflammation. Glial cells include astrocytes, oligodendrocytes, and microglia, and inflammatory mediators released by astrocytes and microglia are central to brain inflammatory responses (Olude et al., 2022). VWR can affect the CNS immune environment and reduce overactivation of glial cells, especially astrocytes and microglia (Spielman et al., 2017). In one study, the number of microglia in the cerebral cortex decreased significantly after 6 weeks of VWR in C57BL/6 J and Thy1-GFP mice (He et al., 2017). Changes in microglia were also observed in rats with traumatic brain injury. Microglial activation density was much higher after brain trauma, whereas the expression of microglial markers, including iNOS, CD16, and IL-1*β*, significantly decreased after VWR (Hu et al., 2023).

Neuroplasticity is the ability of the nervous system to adapt to external stimuli. Exercise can increase neuronal connectivity, neurotransmitter release, and neurotrophic support, thereby reducing pain signaling associated with neuroinflammation. VWR can reduce memory impairment after hippocampal ischemia by decreasing inflammation and apoptosis (Farokhi-Sisakht et al., 2020). VWR can also reverse memory impairment in an NP model by increasing IL-10 and limiting the negative effects of IL-1*β* or lipopolysaccharide (LPS) on long-term potentiation (Kelly et al., 2001; Lynch et al., 2004; Kiyota et al., 2012; Reguilón et al., 2020). Eadie et al. found that VWR increased the overall length and complexity of dendrites in the mouse dentate gyrus, along with increased spine density, suggesting enhanced hippocampal plasticity (Eadie et al., 2005). The activation of nuclear factor E2-related factor 2 (Nrf2) induces antioxidant enzymes and can protect vessels and neurons against oxidative stress. In a VWR-sedentary comparison, phosphorylated Nrf2 expression was higher in brain capillaries from VWR mice, supporting a role for VWR-induced antioxidant signaling in neuroplasticity (Toborek et al., 2013). Exercise also promotes the expression of brain-derived neurotrophic factor (BDNF) through the ketone body *β*-hydroxybutyrate (Sleiman et al., 2016). Voluntary locomotion can increase BDNF levels (Galvan and Wichmann, 2007), and VWR has been reported to increase BDNF and enhance hippocampal plasticity (Cotman et al., 2007; Iqbal et al., 2026). A comparative study found that after 39 days of VWR, hippocampal BDNF levels were higher in the VWR group than in the sedentary and forced-exercise groups (Griesbach et al., 2014).

### 3.2 Effects of VWR on spinal cord neuroinflammation

NP induces persistent inflammation in the spinal cord and increases cytokines, including TNF-*α* and IL-1β, as well as microglia and astrocytes in the dorsal horn (Krames, 2014; Takahara-Yamauchi et al., 2021). VWR inhibits spinal cord neuroinflammation and may enhance analgesic effects in NP. VWR intervention can downregulate inflammatory factors such as IL-1β in the spinal cord and significantly improve pain behavior (Grace et al., 2016). In EAE mice, 30 days of VWR decreased the pro-inflammatory cytokine IL-17 in the spinal cord and increased Nrf2 and IL-10 expression, indicating that VWR can regulate spinal cytokine balance and improve neuroinflammation (Saffar Kohneh Quchan et al., 2020). Mifflin et al. found that after MOG35-55-injected mice underwent VWR for 30 days, spinal inflammatory markers decreased, pain-related behavior improved, and EAE onset was delayed (Mifflin et al., 2017). Although relatively few studies have directly examined spinal cytokine regulation by VWR, available evidence suggests that VWR can suppress pro-inflammatory signaling and enhance anti-inflammatory responses.

Takahara-Yamauchi et al. reported that VWR reduced hyperalgesia and mechanical hypersensitivity in mice with persistent NP (Takahara-Yamauchi et al., 2021). VWR may relieve neuroinflammation by slowing microglial proliferation, thereby contributing to its analgesic effect. Sánchez-Ventura et al. established an SCI model and compared environmental enrichment, VWR, and forced treadmill exercise (Sánchez-Ventura et al., 2021). Only mice that ran on the voluntary wheel showed significantly improved BMS scores, suggesting that VWR may support spinal reflex modulation and locomotor recovery after SCI. VWR also reduced the astrocyte marker GFAP and the microglial marker Iba1 in the ventral horn. In a formalin-injection experiment, numerous spinal microglia were observed in sedentary mice after 7 days, whereas Iba1 immunoreactivity in the anterior horn and intermediate spinal cord was not observed in the VWR group (Takahara-Yamauchi et al., 2021). Overall, VWR reverses mechanical hyperalgesia and reduces NP by inhibiting astrocyte and microglial activation in the spinal cord.

### 3.3 Effects of VWR on DRG neuroinflammation

Axonal injury in the DRG usually leads to neuronal overexcitation and contributes to NP (Paddock et al., 2018). Nociceptive DRG neuron excitability also increases in rodent models of inflammatory pain (Zhang et al., 2017; Matsuda et al., 2019; Ma et al., 2024). NP can induce abnormal ion-channel activation in the DRG and promote the release of inflammatory factors and chemokines, thereby aggravating pain (Kawai et al., 2018; Sun et al., 2018). Ye et al. (2018) found that VWR combined with chronic ddC treatment relieved mechanical pain sensitivity and reduced nociceptive neuron membrane excitability in the DRG. Related studies indicate that VWR may stimulate axon regeneration and gene-expression changes in DRG neurons (Molteni et al., 2004; Paddock et al., 2018). Glial activation in the DRG produces inflammatory markers and upregulates pro-inflammatory cytokines and chemokines, which are hallmarks of neuroinflammation (Zhang et al., 2017; Matsuda et al., 2019). In CCI rats, 6 weeks of VWR significantly reduced the macrophage marker Iba1, the chemokine CCL-2, and activating transcription factor 3 (ATF3) in the DRG (Grace et al., 2016). ATF3 is a surrogate marker of neuronal damage after peripheral nerve injury (Tsujino et al., 2000), suggesting that VWR may regulate DRG cytokines and ameliorate neuronal injury. The primary sensory neurons of the DRG are encapsulated by satellite glial cells (SGCs), and neuron-SGC interactions influence somatosensory and nociceptive transmission (Chen et al., 2022). Neuroinflammation may activate SGCs and induce pathological changes that contribute to NP (Feldman-Goriachnik et al., 2022). In LPS-injected mice, 7 days of VWR inhibited GFAP upregulation, indicating reduced SGC activation and improved DRG neuroinflammation (Feldman-Goriachnik et al., 2022). Accordingly, VWR may alleviate NP by inhibiting SGC activation in the DRG.

### 3.4 Effects of VWR on other peripheral nerve inflammations

Peripheral nerve injury can cause significant microglial proliferation in the spinal dorsal horn, and the resulting neuroinflammation can contribute to NP involving damaged neurons (Guan et al., 2016; Ehmedah et al., 2020). Numerous inflammatory factors are present in the sciatic nerves of NP animal models, and these inflammatory mediators importantly affect NP occurrence (Kiguchi et al., 2013; Grace et al., 2016). After peripheral nerve injury, inflammatory factors can sensitize peripheral nociceptive neurons directly or indirectly. For example, the pro-inflammatory cytokine TNF-*α* may drive peripheral nerve inflammation, whereas the anti-inflammatory cytokine IL-10 can regulate sciatic nerve inflammation and promote nerve regeneration (Siqueira Mietto et al., 2015; Wang et al., 2022). Exercise training can regulate chronic constriction injury of the sciatic nerve, thereby reducing peripheral nerve pain and excessive TNF-α and IL-1*β* expression (Chen et al., 2012). Sartori et al. administered VWR to C57BL/6 J male mice for 14 days and found that VWR prevented persistent hyperalgesia and even reversed hyperalgesia induced by sciatic nerve ligation (Sartori et al., 2020). Grace et al. found that VWR reduced CCL-2 and Iba1 expression in the sciatic nerve and increased serum levels of anti-inflammatory mediators, thereby improving pain behavior in CCI rats (Grace et al., 2016).

## 4 Potential upstream molecular pathways of VWR-mediated analgesia

Although most VWR studies describe downstream inflammatory changes, several upstream signaling pathways may link voluntary exercise to neuroinflammatory modulation in NP. First, VWR may activate neurotrophic signaling. Exercise-induced BDNF can engage TrkB and CREB-related plasticity pathways, supporting synaptic stability and inhibitory control of nociceptive transmission. Sleiman et al. reported that exercise promotes BDNF expression through the ketone body *β*-hydroxybutyrate, providing a mechanistic link between physical activity, metabolism, and neuroplasticity (Sleiman et al., 2016). In pain models, VWR has also been associated with Nrf2-related antioxidant signaling and reductions in IL-1β, TNF-α, NLRP3, NF-κB p65, Iba1, CCL-2, and ATF3 (Grace et al., 2016; Green-Fulgham et al., 2022; Saffar Kohneh Quchan et al., 2020). These observations suggest that VWR may attenuate peripheral and central sensitization by coordinating neurotrophic, antioxidant, and cytokine-related pathways rather than by suppressing a single inflammatory mediator.

In addition, β-hydroxybutyrate may be relevant beyond energy metabolism because it can act through hydroxycarboxylic acid receptor 2 (HCAR2/GPR109A), a receptor implicated in neuroimmune regulation and pain-related neuroinflammation (Boccella et al., 2019; Taing et al., 2023; Testai et al., 2025). Boccella et al. demonstrated that the β-hydroxybutyrate-HCAR2 axis is involved in neuropathic pain plasticity, whereas recent reviews emphasize that HCAR2 activation can suppress microglial reactivity, reduce pro-inflammatory cytokine release, and modulate neuronal excitability (Boccella et al., 2019; Testai et al., 2025). Therefore, future studies should directly test whether VWR-induced metabolic changes regulate microglia, astrocytes, SGCs, and macrophage responses through BDNF/TrkB-CREB, β-hydroxybutyrate/HCAR2, Nrf2, and NF-κB/NLRP3 signaling. Clarifying these upstream pathways would strengthen the translational value of VWR as a non-pharmacological intervention for NP.

## 5 Conclusion

This review summarizes VWR-induced neuroinflammatory changes in NP and the potential mechanisms by which VWR alleviates pain. VWR decreases pro-inflammatory cytokines, increases anti-inflammatory mediators, downregulates microglia and astrocyte markers such as Iba1 and GFAP, inhibits SGC activation in the DRG, and reduces macrophage-related inflammation in peripheral nerves. These effects suggest that VWR alleviates NP symptoms by modulating neuroinflammation across multiple regions of the nervous system. Mechanistically, VWR may act through coordinated neurotrophic, metabolic, antioxidant, and cytokine-related pathways, including BDNF/TrkB-CREB, β-hydroxybutyrate/HCAR2, Nrf2, and NF-κB/NLRP3 signaling. Future studies should directly test these upstream pathways and clarify how VWR-induced molecular changes translate into stable analgesic effects in NP. Within a broader biopsychosocial framework of chronic pain, VWR may also have translational relevance as a non-pharmacological strategy that links biological mechanisms with behavioral modulation.
