# Correction: Voluntary wheel exercise reduces pain behavior by modulating neuroinflammation in neuropathic pain

**DOI:** 10.3389/fnmol.2026.1920423

**Published:** 2026-08-05

**Authors:** Yuqi Zhu, Wenyang Xu, Jinhui Zhang

**Affiliations:** 1 School of Economics and Management, Shanghai University of Sport, Shanghai, China; 2 School of Exercise and Health, Shanghai University of Sport, Shanghai, China; 3 Department of Physical Education, Xidian University, Xi'an, China

**Keywords:** analgesic effect, analgesic mechanism, neuroinflammation, neuropathic pain, voluntary wheel running

There was a mistake in Figure 1 as published. In Figure 1, Interferon-gamma was erroneously abbreviated as INF-γ, whereas the globally accepted standard scientific nomenclature is IFN-γ. In addition, there is a cell icon labeled SCGs. In the context of the dorsal root ganglion, these glial cells are known as Satellite Glial Cells, which should be abbreviated as SGCs. The corrected Figure 1 appears below.

**Figure 1:**
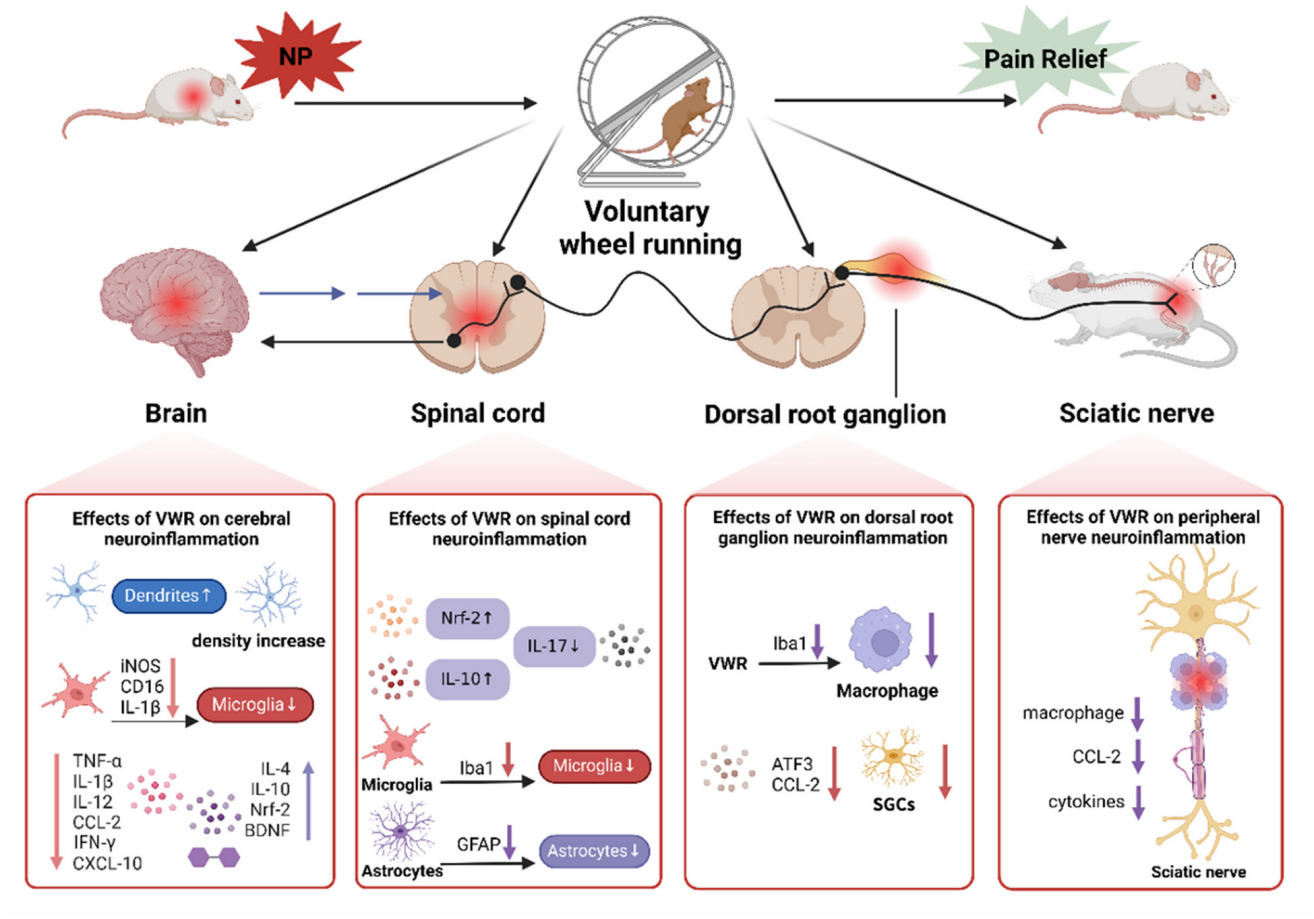
The mechanisms involved in the relief of neuropathic pain by voluntary wheel running. Created with BioRender.com. VWR, voluntary wheel running; IL-17, interleukin-17; IL-10, interleukin-10; IL-6, interleukin-6; IL-4, interleukin-4; IL-1β, interleukin-1β; BDNF, brain-derived neurotrophic factor; TNF-α, tumor necrosis factor-α; IFN-γ, interferon-γ; Nrf2, nuclear factor E2-related factor 2; ATF3, activating transcription factor 3; CCL-2, C-C motif chemokine ligand 2; GFAP, glial fibrillary acidic protein; SGCs, satellite glial cells; DRG, dorsal root ganglion; NP, neuropathic pain.

The original version of this article has been updated.

